# Health-Promoting Behavior and Lifestyle Characteristics of Students as a Function of Sex and Academic Level

**DOI:** 10.3390/ijerph19127539

**Published:** 2022-06-20

**Authors:** Carsten Müller, Kareem El-Ansari, Walid El Ansari

**Affiliations:** 1 Department of Applied Health Sciences, Hochschule für Gesundheit, 44801 Bochum, Germany; carsten.mueller@hs-gesundheit.de; 2 University Sports, University of Münster, 48149 Münster, Germany; 3 Faculty of Medicine, Ain Shams University, Cairo 11591, Egypt; kareemelansari@gmail.com; 4 Department of Surgery, Hamad General Hospital, Hamad Medical Corporation, Doha 3050, Qatar; 5 College of Medicine, Qatar University, Doha 2713, Qatar; 6 Weill Cornell Medicine—Qatar, Doha 24144, Qatar

**Keywords:** college students, gender, sedentary behavior, resistance training, healthy diet, sleep quality, smoking, neuroenhancement, tobacco, alcohol

## Abstract

University students frequently engage in unhealthy behaviors. However, there is a lack of studies examining a wide range of their lifestyle characteristics by sex and academic level of study. This cross-sectional survey of students enrolled in BSc, MSc, or PhD programs at one university in Germany (*N* = 3389) assessed physical activity (PA), sedentary behavior (SB), nutrition, sleep quality, and alcohol, tobacco, and other drug (ATOD) use by sex and academic level and was conducted with EvaSys version 8.0. Chi-squared tests compared categorical variables by sex, and binary logistic regression analyses adjusted for sex with Bonferroni adjustments evaluated differences across academic level. Although 91% of students achieved the aerobic PA guidelines, only 30% achieved the muscle strengthening exercises (MSE) guidelines, and 44% had high SB. Likewise, <10% met the fruit and vegetable consumption (FVC) recommendations, >40% of students experienced impaired sleep, and >30% had hazardous alcohol consumption. Less than 20% of the sample achieved the guideline/recommendation of all three PA, MSE and SB. Some behaviors exhibited significant sex and academic level differences. The identified at-risk groups included males (lower FVC), females (eating more during stress), and BSc students (poorer nutrition/sleep quality, more ATOD use). Given the above findings, multipronged strategies are needed with an overarching focus highlighting the health–academic achievement links. Behavioral interventions and environmental policies are required to raise awareness and promote student health.

## 1. Introduction

The period of university study represents many new challenges for emerging adults, including organization of everyday life, studies, and social environment, as well as taking responsibility for one’s own health during a period where one is generally assumed to be in good to very good health [1]. Such circumstances are more challenging for younger freshmen and sophomores who are less experienced with the healthcare system and the health-promotion resources in their environment [2]. Hence, the university period is frequently accompanied by new unhealthy practices and routines that could impact students’ health and lifestyles into adulthood, which is crucial as behavioral modifications are more difficult to implement in later life [1,3,4]. For instance, most students with sufficient physical activity (PA) levels at college were sufficiently physically active six years after graduation, while most students with insufficient PA levels remained inactive [5]. Lifestyles characterized by adequate PA, nutrition, sleep, and no substance use help to maintain physical and mental health and reduce the risk of non-communicable diseases [6].

However, published reports found that large proportions of university students do not meet the current PA guidelines and sedentary behavior recommendations, e.g., in Finland, the USA and the UK [7,8,9]. Moreover, universities represent a setting where students spend large amounts of time sitting, as measured by self-reports and accelerometry [10,11]. This might prove detrimental for academic performance and overall health, as PA and sedentary behavior (SB) are significantly associated with quality of life [12], perceived health [13], or stress, anxiety, and depression [12,14,15]. Of note, sex differences have been reported in terms of PA and SB [12], as well as significantly increased sitting time from freshmen to senior years [16]. Sex differences have also been observed among university students regarding their achievement of dietary recommendations, e.g., in the UK, Nigeria, Greece, China and Finland [9,17,18,19,20], or their food choices during stressful periods [21,22], yet no firm conclusions could be drawn whether females or males consume healthier diets. Nevertheless, fruit and vegetable consumption (FVC) appear to be significantly associated with cardiovascular and mental health in otherwise healthy populations [23,24].

Restful sleep is another important component of health behaviors. Among university students, sleep quality and quantity are closely associated with student learning capacity and may impact their academic performance [25,26]. Globally, previous research reported poor sleep among university students [27,28,29], and significantly impaired sleep quality compared with the general population [30]. For instance, severe sleep problems have been shown in Italy, with one in four students reporting nocturnal symptoms of insomnia [30]. In the United States, 62% of college students met the cutoff criteria for poor sleep with a higher prevalence of impaired sleep among females [31]. Likewise, an Ethiopian study reported poor sleep quality in 56% of the student sample, with significantly higher odds for females, and lower odds for senior students (fourth semester) compared with third semester and sophomore students [32]. Indeed, poor sleep among college students including sex differences has been reported across the globe [32,33,34,35,36,37].

Likewise, alcohol, tobacco and other drugs (ATOD) can seriously interfere with academic performance at university [38,39]. A recent report from Finland found a high prevalence of smoking, alcohol consumption and other substance use among university students [40]. Likewise, the prevalence of ATOD use among college students ranged between 41–70% in Kenya, Oman, and India [41,42,43]. Similarly, in the UK, the level of binge drinking and problem drinking was high among students, and males generally reported higher use of tobacco, illicit substances, and alcohol [9].

However, we are not aware of larger-scale studies considering multiple health behaviors in university students in Germany. Most of the larger-sample-sized studies focused on single behaviors like nutrition [44], sleep [45], or ATOD [46], whereas studies on physical activity levels are generally scarce (e.g., [47]), thus producing biased estimates. Moreover, previous reports examining multiple health behaviors are outdated [48], whereas more recent studies were carried out with smaller samples (*N* < 1 k students) [49,50]. Furthermore, as universities are increasingly encouraged to promote a healthy study environment with only limited resources, a solid and continuously updated database regarding student health and wellbeing is required to derive targeted and sustainable health promotion interventions and strategies. That said, a differentiation of health behaviors by gender and academic level may serve as an initial step in identifying particularly vulnerable university students as a target group for health interventions.

This study tries to bridge these research gaps by extensively describing multiple health behaviors associated with overall student health and academic performance as a function of gender and academic level comprising BSc, MSc, and PhD-students. Our four specific objectives were to assess:

1. PA, i.e., aerobic PA levels and muscle strengthening exercises (MSE) according to current World Health Organization (WHO) guidelines, as well as SB and self-rated fitness level;
2. Nutrition, i.e., importance of eating healthily, average FVC, cooking/self-catering, eating habits during stressful periods, and water intake;
3. Sleep quality, i.e., overall sleep impairment, latency, duration, efficiency, disturbances, and daytime dysfunctions;
4. ATOD, as well as self-rated perception of hazardous alcohol consumption.

## 2. Materials and Methods

### 2.1. Ethics, Study Design, Sample, and Procedures

The study was approved by the University Ethics Committee (approval # 2019-07-TU). This online cross-sectional survey was conducted during the 2019 summer term at the University of Münster (WWU), the fifth largest university in Germany, comprising 15 faculties and 21 departments, covering social sciences (e.g., psychology, sociology, political science), natural sciences (e.g., physics, chemistry, biology), and humanities (e.g., religion, philosophy, linguistics).

All regular students (*N* = 42,630) received email invites. Addresses were provided by the university administration. Therefore, no a priori sample size calculation was performed. Invitations included information about the study background and objectives, time required to complete the questionnaire (~20–30 min), the voluntary nature of participation, anonymity, and privacy. Participants completed an online informed consent before commencing the survey. The questionnaire was provided in validated German and English language versions. Code numbers ensured that students could participate only once. Non-participants received two e-mail reminders. Given the scope of the study, the present analyses included only the responses from Bachelor of Science (BSc), Master of Science (MSc), and doctoral (PhD) students (*N* = 3389). For other degrees (e.g., state exam, diploma, *N* = 855), no differentiation can be made between undergraduate and graduate students. We employed the software EvaSys version 8.0 (Electric Paper Evaluationssysteme GmbH, Lüneburg, Germany), a web-based software for the automation of surveys, examinations, and for the support of quality management in studies and teaching, allowing for adaptive questioning and plausibility checks.

### 2.2. Data Collection

#### 2.2.1. Physical Activity

PA levels were assessed using the short form of the International Physical Activity Questionnaire (IPAQ-SF) for adults aged 15–69 years [51]. The IPAQ-SF had acceptable measurement properties in adult populations as well as university students, in whom a 77% agreement with accelerometer-determined compliance to the PA guideline and a moderate test–retest reliability (ICC = 0.52) was demonstrated [51,52,53]. The questionnaire is available in English and German languages [54]. The IPAQ-SF asks about daily walking, moderate, and vigorous aerobic PA, comprising activities such as running, cycling, and swimming, referring to the previous week (Table 1). The frequency (number of days) and duration (10–180 min/day) of each of these activities was assessed, allowing the calculation of weekly metabolic equivalent of task minutes (MET-min/week), and subsequently the corresponding PA level (low, moderate, high). MET-min/week are calculated by multiplying frequency × duration × intensity, with intensity referring to the average MET estimate for a given activity (walking = 3.3, moderate PA (MPA) = 4, vigorous PA (VPA) = 8) [51]. The IPAQ-SF also asks about daily sitting time in hours. Likewise, the frequency of MSE was assessed by asking “On how many of the last seven days did you participate in strength training of ≥10 min (e.g., strength training with your own body weight, strength training with gym equipment)?” [55].

Based on current (inter)national recommendations, adults should perform ≥ 150 min of MPA, or ≥75 min of VPA, or an equivalent combination of moderate to vigorous PA (MVPA) throughout the week [56,57]. Furthermore, adults should undertake MSE on ≥2 days/week, and limit the amount of sedentary time [56,57]. Once a total of ≥600 MET-min/week is accumulated, the aerobic PA guidelines are met. Given that the PA guidelines do not comprise a cutoff for daily sitting time [58], a threshold of ≤8 h/day was set to differentiate between achieving and not achieving the SB recommendations. This threshold is derived from a recent meta-analysis where prolonged sitting > 8 h/day was associated with increased cardiovascular disease and cancer mortality [59]. Finally, the MSE guidelines were accomplished if students indicated ≥ 2 MSE sessions/week.

Self-rated fitness was assessed by asking “How do you rate your own physical fitness?” on a five-point Likert scale, ranging from “very good” to “very poor” [60]. For the current analysis, these were collapsed into two options: very good/good vs. poor/very poor.

#### 2.2.2. Nutrition

Students’ nutritional behavior was assessed in relation to the importance of eating healthily, diet habits, and eating behavior during stressful times. The importance of a healthy diet was rated on a five-point Likert scale (very unimportant to very important) [20]. Participants also rated the amount of their daily FVC, using a four-point Likert scale ranging from “I do not eat vegetables/fruit” to “≥5 servings/day”, corresponding to the current national and international recommendations [61,62]. Additionally, we asked “How many times per week do you prepare your meals yourself?” using 1 = not at all, 2 = 1–2 times, 3 = 3–4 times, 4 = 5–6 times, and 5 = daily [63]. Given any probable stress-induced changes in food choices, students rated how much they agreed to the statement “In very stressful periods, I generally eat…”, on a five-point Likert scale (1 = significantly less, 5 = significantly more, later collapsed into the three categories “somewhat/ significantly less” vs. “unchanged” vs. “somewhat/ significantly more”.

Sufficient fluid intake was assessed by the question: “How much fluid do you consume on average through water per day” with the anchors 1 = <1 L per day (L/d); 2 = 1–1.5; 3 = 1.5–2; 4 = 2–2.5; 5 = 2.5–3, and 6 = >3 L/d. Students were also asked to indicate their current body weight in kg. Given sex-specific differences for adequate intake [64,65], we approximated water consumption based on body mass using the formula:

$$\mathrm{water}\;{\mathrm{intake}}_{\mathrm{bm}}=\frac{\left(\mathrm{anchor}\times 0.5\right)+0.25}{\mathrm{body}\;\mathrm{mass}}.$$

In line with the German Nutrition Society (DGE) water consumption recommendations, adequate intake was set at ≥30 mL/kg body mass [66].

#### 2.2.3. Sleep Quality

We assessed sleep quality and patterns using the short-form Pittsburgh Sleep Quality Index (sPSQI), a 13-item questionnaire that evaluates sleep within the past four weeks and comprises five dimensions (sleep latency, duration, efficiency, disturbances, and dysfunction). Derived from the validated 19-item PSQI that discriminates between “good” and “poor” sleepers (sensitivity of 90% and specificity of 87%) and has a high degree of internal consistency (Cronbach’s alpha = 0.83) [67], the shortened version correlates well with the original PSQI among college students (rho = 0.94, *p* < 0.001), but with the advantage of reduced respondent burden [68]. Lower scores indicate better sleep, and a score ≥ 5 is indicative of impaired sleep quality.

#### 2.2.4. Alcohol, Tobacco, and Substance Use (ATOD)

Alcohol consumption was assessed using the short version of the Alcohol Use Disorders Identification Test, AUDIT-C [69], as recommended by current German S3-guidelines on screening for hazardous alcohol consumption or alcohol dependence [70]. The questionnaire assesses the frequency of alcohol consumption and binge drinking behavior using three items (scores range from 0–12). Hazardous drinking behavior is indicated by a sum score of ≥4 in females and ≥5 in males, reflecting the increased vulnerability to alcohol-related harm in women. These cutoffs demonstrated an optimal balance of sensitivity (females 0.81, males 0.80) and specificity (females 0.86, males 0.93) across student samples from different universities in Germany [71]. In addition, students self-rated their alcohol consumption using the question “My alcohol consumption is harmless” on a five-point Likert scale (1 = fully applies, 4 = does not apply at all, 5 = cannot judge). Smoking status was assessed by asking “Do you smoke?” with a dichotomous response (yes/no) and examples were presented: cigarettes, e-cigarettes, cigars, cigarillo, pipe, or hookah. If the answer was “yes”, the questionnaire further asked, “Do you smoke daily?” [72].

Substance use was assessed using two items with dichotomous response options: “Since the beginning of your studies, did you consume substances that would help overcoming the requirements of your study program (e.g., sedatives or substances improving efficiency)?” [73]. Examples were provided: psychotropic drugs (e.g., valium, soporifics, sedatives), cannabis, amphetamines (speed, ecstasy), cocaine, prescription painkillers, methylphenidate (Ritalin), vitamin products, energy drinks, antidepressants, caffeine tablets. If the answer was “no”, we further asked “During your studies, have you ever thought of the consumption of substances which would help overcoming the requirements of your study program (e.g., sedatives or substances improving efficiency)?” [73].

Table 1 summarizes the definitions and (inter)national guidelines/recommendations of the variables under study.

**Table 1:** Definitions and international guidelines/recommendations of terms used.

Behavior	Definitions and International Guidelines/Recommendations
PA	**D:** Any bodily movement produced by skeletal muscles that requires energy expenditure [74]
MPA	**D:** e.g., carrying light loads, bicycling at ordinary speed, or swimming at ordinary speed
VPA	**D:** e.g., aerobic exercise, running, fast cycling or fast swimming
MVPA	**D:** Moderate to vigorous intensity PA
Aerobic PA	**G:** Adults should do ≥150 min of MPA; or ≥75 min of VPA; or an equivalent combination of MVPA throughout the week, for substantial health benefits [56]
SB	**D:** For adults, time spent sitting or lying with low energy expenditure, while awake, in the context of occupational, educational, home and community settings, and transportation [56] **R:** SB <8 h per day, as recent meta-analysis reported that adults sitting for >8 h/day had a higher risk of CVD and cancer mortality [59]; no official guideline available, as evidence is insufficient to quantify a SB threshold [58]
MSE	**G:** Adults should also do muscle-strengthening activities at moderate or greater intensity that involve all major muscle groups on ≥2 days a week, as these provide additional health benefits [56]
FVC	**R:** ≥5 servings/day [61,62]
Water intake	**R:** ≥30 mL/day per kilogram body mass [66]
Sleep	**R:** The appropriate sleep duration for young adults is ≥7 h [75]
Alcohol	**G:** The tolerable upper alcohol intake levels have been set at 10–12 g/day for healthy women and 20–24 g/day for healthy men of the adult population [76]

PA: Physical activity; MPA: Moderate PA; VPA: Vigorous PA; SB: Sedentary behavior; MSE: Muscle strengthening exercises; FVC: Fruit and vegetable consumption; D: definition; G: guideline, R: recommendation.

### 2.3. Statistical Analysis

Categorical variables are presented using frequency (percentage), while quantitative variables are presented as mean ± standard deviation. The chi-squared test compared the samples for any sex differences across the categorical variables. Differences based on the academic degree pursued were compared using binary logistic regression analyses adjusted for sex and with Bonferroni adjustments for multiple group comparisons. Analyses were performed using IBM SPSS v.28 (IBM Corporation, Armonk, NY, USA), and the statistical significance level was set at *p* < 0.05.

## 3. Results

The overall response rate was 10%. Students from across all university departments participated in the survey, and participation rates among the various departments ranged from 7–22%, as some students were enrolled in more than one department at the time of data collection. About 67% of the respondents were female.

### 3.1. Health Behavior and Lifestyle Characteristics by Sex

#### 3.1.1. Physical Activity and Self-Rated Fitness

The students reported a mean 2452 ± 1989 MET-min/week, with 1146 ± 1354 and 672 ± 747 MET-min attributable to VPA and MPA, respectively. Another mean 634 ± 831 MET-min were derived from walking. Using the IPAQ-SF classification, slightly less than half the sample was categorized as highly physically active (Table 2). In terms of aerobic PA, >90% of the respondents achieved the WHO guidelines. Average sitting time was 9.3 ± 5 h per day, and about 56% of the sample reported sitting < 8 h/day, in line with the recommendation on SB. MSE were performed on average 1.3 ± 1.5 days/week, and slightly less than one out of three students achieved the current MSE guidelines. Only 19% of the sample met all the three PA recommendations.

Significantly higher proportions of males met high PA levels according to the IPAQ-SF classification and met the MSE guidelines. Conversely, significantly more females met the aerobic PA guideline and SB recommendation. There was no sex difference regarding achieving all 3 PA recommendations. Despite this, significantly more males rated their fitness level as very poor/poor.

#### 3.1.2. Nutrition

Slightly more than a third of the sample indicated that eating healthily was very important, yet only 8% consumed ≥ 5 servings of vegetables/fruits per day and thus complied with the recommendations (Table 3). Almost one in five students prepared their own meals daily. Noticeably, >77% of the respondents altered their eating habits when stressed, with 33% and 44% of students eating less/significantly less or eating more/significantly more, respectively. Regarding water consumption, about one quarter of the sample drank sufficient water according to current recommendations. Generally, we observed sex differences across the nutrition variables. More females rated eating healthily as very important, achieved the recommendation of daily FVC, and self-prepared meals daily. However, significantly more females ate somewhat/significantly more during stressful periods. The proportions of students reporting sufficient water intake did not differ by sex.

#### 3.1.3. Sleep Quality

Impaired sleep quality was reported by 42% of the student sample (Table 4). Subscale analyses indicated very long sleep latency (5–6 h) in almost 10% of the students. Very short sleep durations of ≤6 h were reported by 6%, and low sleep efficiency of <75% was prevalent among 9% of the respondents. Less than 10% experienced ≥ 7 weekly sleep disturbances, whereas 40% had ≥3 daytime dysfunctions within the previous week. Overall sleep quality did not differ significantly by sex. However, (very) long sleep latencies, and higher amounts of sleep disturbances and daytime dysfunctions during the previous week were more common among females.

#### 3.1.4. Substance Use

In terms of ATOD, more than one third of the sample reported hazardous alcohol consumption, whereas more than three out of five students rated their alcohol consumption as harmless (Table 5). Nearly 10% of the participants smoked occasionally or daily. In terms of substance use, almost 14% previously used substances to cope with study demands, and another 22% had thought about using substances to cope with study demands since the beginning of their studies. No significant sex differences were found regarding hazardous alcohol consumption or substance use, although significantly more females rated their alcohol consumption as harmless, and higher proportions of males smoked.

### 3.2. Health Behavior and Lifestyle Characteristics by Academic Degree Pursued

Few health behaviors differed significantly by academic level (Table 6). The proportion of PhD students achieving aerobic PA guidelines was significantly lower compared with BSc and MSc students, whereas significantly more MSc students reported SB in accordance with current recommendations. Furthermore, fewer BSc students reported a healthy diet as rather/very important compared with MSc respondents, whereas the percentage of students who simultaneously reported recommended FVC and water intake was highest in PhD students. As for sleep, lower academic level was associated with a higher prevalence for impaired sleep. Conversely, significantly more BSc students smoked occasionally or daily compared with PhD students. A higher proportion of BSc compared with PhD students also thought about using or used substances to cope with study demands. Collectively, except for PA, the findings suggest that the percentage of students with the least healthy behaviors was lowest in BSc students compared with those attending higher academic levels.

## 4. Discussion

This study assessed student health behaviors by gender and academic level. The main findings were that <20% met the guidelines for minimum PA level, with no sex/academic level differences. Eating healthily was important for most respondents, although <10% of students met the FVC recommendations. Over 40% of students experienced impaired sleep (no sex differences), and more BSc students had impaired sleep compared with other academic levels. More than one-third of participants had hazardous alcohol consumption, with no sex differences. More males smoked occasionally/regularly, and significantly more BSc students were smokers/substance users than PhD students.

Most of our students achieved the guidelines for aerobic PA (>600 MET min/week), in line with other reports from Ireland [53]. This might support a viewpoint that the IPAQ-SF threshold that distinguishes between achieving and not achieving aerobic PA guidelines might be relatively low [53,77]. The facilities and faculties of the University of Münster are spread throughout the city, requiring regular commuting that might have contributed to why most students met the aerobic PA guidelines. A higher proportion of our females met the aerobic PA guidelines, contrary to others who found no sex differences [78] or higher aerobic PA among males [53,79]. The university sports center offers a very large variety of non-competitive sports, which are usually more appealing to females and might have contributed to their higher aerobic PA rates.

Pertaining to MSE, 30% of our sample achieved the guidelines (no sex difference), higher than the UK (19%), but lower than the USA (48%) [9,80]. However, other research found a significant male predominance in meeting the MSE recommendations (41%_female_ vs. 51%_male_) [78] or (20%_female_ vs. 36%_male_) [81], probably due to males’ higher intentions and self-efficacy, known to be associated with concordance to MSE guidelines [82,83]. Notwithstanding, more of our females reported occasional MSE than males.

As for SB, 17% and 39% of the current sample reported low and moderate sitting times, respectively. The remaining 44% had high SB, recognized to be linked to increased morbidity/mortality [59,84]. Generally, university students spend much time sitting, exceeding the SB of the general young adult population [10]. Nevertheless, our mean SB (9.3 h/day) exceeded the mean sitting time of college students reported in a recent meta-analysis (7.3 h/day) [10]. Fewer of our PhD students met the SB recommendations compared with MSc/BSc students, supporting that SB increases with higher academic degrees [60].

When considering the achievement of aerobic PA and MSE guidelines together with the SB recommendations, <20% of the current students achieved all three. Comparisons with other research are challenging as previous studies either examined student achievement of the recommendations for PA individually, or for PA and MSE, but did not appraise the combination of all three together (PA, MSE, and SB) (e.g., [81,85,86,87]).

Nutrition patterns significantly impact on health, and high FVC provides vitamins, minerals, fiber, and low calories. About 79% of the current sample viewed eating healthily as very/rather important, identical to Finland (79%) [20]. Likewise, the significant sex differences among our students who rated healthy eating as important (82%_female_, 70%_male_) resembled Finland (83%_female_, 69%_male_) [20]. Despite attaching high importance to healthy eating, <10% of the current students met the recommendations of ≥5 servings/day, slightly lower than among young adults in the general German population [61]. We also observed sex differences in healthy nutrition (52%_female_ vs. 32%_male_ consumed ≥ 3 servings of vegetables/fruits per day), concurring with Finland, Nigeria, and China [17,19,20].

Home prepared meals offer multiple benefits, e.g., eating smaller portions, healthier foods (less fat, salt, sugar, cholesterol, calories), and is linked to higher probabilities of not eating fast food as well as meeting FVC and nutrient goals [63,88,89]. About one fifth of our students cooked daily, with sex difference in the proportion of those who prepared their own meals on ≥5 days/week (45%_female_ vs. 35%_male_). Our findings are comparable to the USA, where 41%_female_ and 24%_male_ prepared their own meals on most days [90].

Students have higher stress levels compared with non-students, females are more stressed than males, and stress impacts directly on psychological/physical health and indirectly modifies food choices [91,92,93]. We found sex differences among participants who ate more during periods of stress (51%_female_ vs. 31%_male_), in line with the predominance of female students reporting increased meal sizes and less healthy food choices during stress [22,94,95].

Insufficient water intake is negatively associated with cognitive performance, attention, psychomotor, and immediate memory skills among young adults [96]. With no official guideline for water intake, the national DGE recommendation for young adults (2.7 L/day) falls in between the USA recommendations (Institute of Medicine) and European guidelines (Food Safety Authority) [64]. Using the national DGE recommendation, only 26% of our sample had adequate water intake. This agrees with the lower-than-recommended fluid intake of university students in Iran [97] and that 25% of students had optimal fluid intake in Europe [98].

Impaired sleep was reported by 42% of our respondents, lower than the US (62%), Portugal (68%), and Ethiopia (56%) [31,32,99]. We found that more females experienced high sleep latencies, sleep disturbances, and daytime dysfunctions, supporting similar sex differences in Ethiopia [32]. Impaired sleep is linked to adverse mental health and academic performance [100]. In the USA, there is a consistent increase of students dissatisfied with their sleep [101]. Women report more sleep difficulties [102], and regularly worse subjective sleep quality than men, describing their sleep quality as poor due to night-time disruptions, insufficient quantity, and long sleep latencies [103]. Likewise, research among young women has shown fluctuations in sleep events during the different phases of the menstrual cycle that are associated with the levels of sex steroids [104].

As for ATOD, 5% of the current sample were occasional and 5% were daily smokers, lower than Finland (16% occasional, 6% daily), Italy (33% current smokers), and the UK (12% occasional, 16% daily) [105,106,107]. About 37% of our German students had hazardous alcohol consumption, similar to Finland (33%), but higher than the 16–27% reported in seven European countries [108,109]. We found no sex differences, in contrast to Finland, where males had a higher risk for hazardous alcohol consumption [110]. Although we used the AUDIT-C questionnaire as recommended by the German guidelines, comparisons with other studies were challenging due to the various approaches of assessing alcohol consumption (e.g., time span of recall, cut-offs for hazardous drinking).

Substance use amongst college populations remains a worldwide concern [42,111,112,113,114,115]. About 14% and 22% of our sample reported to have used/thought about using substances to cope with study demands, respectively. In Finland, 1.5% and 19% of the sample regularly and occasionally used illicit drugs, respectively [116]. Whilst we observed no sex differences, male students in the UK were 4.6 and 1.9 times more likely to use illicit drugs regularly or occasionally, respectively [117]. Again, comparison between studies is difficult due to the multiplicity of substances, terms, frequency of use and categorizations employed to group substances [117,118,119].

Academic level was inversely associated with achieving the aerobic PA guidelines. Likewise, our MSc and PhD students differed significantly in meeting the SB recommendations, supporting that SB significantly increased from freshmen to senior students [16]. As for nutritional habits, we found only that significantly more MSc than BSc students considered healthy eating important, and more PhDs met both the FVC and water intake recommendations, concurring with the USA and China, where graduate students rated healthy eating significantly higher, and more frequently achieved the FVC recommendations than undergraduates [19,120]. Academic level was also associated with sleep quality, where more of our BSc than MSc/PhD students had impaired sleep, congruent with Ethiopia [32], and partially supporting Taiwan, where freshmen had shorter sleep duration than seniors, but seniors had higher sleep latencies [36]. Likewise, academic level was associated with substance use, as significantly more BSc than PhDs had used/thought about using substances, concurring with the UK, where younger students were 1.7 and 1.9 times more likely to use illicit drugs regularly or occasionally, respectively, compared with older students [117]. Such findings might propose a cohort effect, suggesting that substance use might have increased over the recent years, or the likelihood of thinking about/using substances decreases as students progress through academic life.

### 4.1. Future Implications

Given the above findings, multipronged strategies need an overarching focus highlighting the health–academic achievement links, e.g., insomnia, excessive alcohol and dehydration that are associated with poorer academic performance and cognition [96,99,121,122,123]. Efforts should consider student participation in all student health promotion processes, target the student body, and particularly the identified risk groups e.g., males (lower FVC), females (eating more during stress), and BSc students (poorer nutrition/sleep quality, more ATOD use). The social norms approach could underpin the interventions [124].

Promoting exercise can focus on increasing MSE and reducing SB, using behavioral (e.g., physically active teaching/learning) and environmental approaches (e.g., PA-promoting campus, advancement and development of the university sports program), while stimulating social unacceptability of SB [125]. Healthy lifestyle efforts need to consider increasing FVC, students’ limited finances and cooking facilities, encourage meal planning/home food preparation, increase knowledge and options regarding water intake (water dispensers/fountains), and increase awareness and coping with sleep problems through information (e.g., sleep lectures) or relaxation interventions (e.g., mindfulness programs) [126,127,128]. Evidence-based face-to-face approaches using motivational interviewing and personalized feedback for hazardous alcohol consumption could prove beneficial [129].

### 4.2. Limitations

This study has limitations. Being a descriptive cross-sectional prevalence study, the direction of effects cannot be ascertained, and generalizations should be cautious. Data were collected at one university and the sample is a convenience sample, which is not uncommon, e.g., in Hong Kong, USA, or Australia [130,131,132]. Self-reports could suffer recall bias, sociability, and social desirability [133], and objective measures would have been beneficial, e.g., cotinine level for tobacco consumption, body composition (fat/muscle) scan for fitness, or estrogen and progesterone as well as melatonin level for sleeping quality. Likewise, “unhealthy”, or “healthy” diets are not absolute concepts, e.g., the ketogenetic diet, although healthy, does not consume fruits or consumes very low amounts of selected fruits. Although the questions regarding dietary behavior and substance use were based on pre-existing questionnaires, no formal test of validity and reliability was conducted and should be considered when interpreting the results. Finally, the low response rate of 10% must be recognized as a limitation. The reasons for this can be seen in the large nature of the survey, with >170 items requiring between 20–30 min, but also in the low interest in a health survey at an age that is predominantly characterized by good health. Despite this, the study has many strengths, including a generous sample of students (*N* = 3389) from across all the university departments/faculties reporting on a wide range of health behaviors pertinent to health and academic performance. Contrary to others, we described both the achievement of recommendations for the individual types of PA, MSE, and SB, as well as their combination. The study used (inter)national questionnaires and recommendations and analyzed data by sex and three academic levels, thus extending previous college health reports that focused on a single/few health behavior(s) among undergraduates.

## 5. Conclusions

Some lifestyle patterns identified in the current study are concerning. Efforts are required to promote PA and healthy nutrition, better sleep quality, and prevent substance use, all of which are associated with academic performance. Universities need to plan and evaluate appropriate strategies based on periodic health reports to motivate healthier lifestyles among their students, encompassing multi-component and evidence-based interventions that ideally combine behavioral and structural preventative measures.

## Figures and Tables

**Table 2:** Physical activity and fitness characteristics by sex.

Characteristic	Total	Female	Male	*p*-Value
	*N* (%)	*N* (%)	*N* (%)	
Physical activity level *^a^*				<0.001
High	1467 (44.5)	968 (43.9)	499 (45.7)	
Low/Moderate	1832 (55.5)	1238 (56.1)	594 (54.3)	
Achieved aerobic PA guidelines *^b^*				0.001
Yes	3013 (91.3)	2040 (92.5)	973 (89.0)	
No	286 (8.7)	166 (7.5)	120 (11.0)	
Sedentary behavior				0.010
Low SB (<6 h/day) *^c^*	564 (17.1)	403 (18.3)	161 (14.7)	
Moderate SB (6–8 h/day) *^c^*	1296 (39.3)	874 (39.6)	422 (38.6)	
High SB (≥9 h/day)	1440 (43.6)	929 (42.1)	511 (46.7)	
Muscle strengthening exercises				<0.001
Achieve MSE guidelines (≥2 times/week) *^b^*	989 (30.0)	642 (29.2)	347 (31.7)	
Occasional MSE (<2 times/week)	611 (18.5)	453 (20.6)	158 (14.4)	
No MSE	1698 (51.5)	1107 (50.3)	591 (53.9)	
Achieve all three PA recommendations *^d^*				NS
Yes	634 (19.2)	417 (18.9)	217 (19.9)	
No	2667 (80.8)	1791 (81.1)	876 (80.1)	
Self-rated fitness level				<0.001
Very good/good	699 (21.2)	465 (21.1)	234 (21.4)	
Fair	1125 (34.1)	804 (36.5)	321 (29.3)	
Very poor/poor	1471 (44.6)	932 (42.3)	539 (49.3)	

All cell values are frequency (%); *p* values based on chi-squared test; *^a^* based on IPAQ-SF categorization; *^b^* based on WHO guidelines; *^c^* recommendation for SB; *^d^* based on achieving aerobic PA and MSE guidelines and SB recommendation.

**Table 3:** Student nutritional behavior by sex.

Characteristic	Total	Female	Male	*p*-Value
	*N* (%)	*N* (%)	*N* (%)	
Importance of eating healthily				<0.001
Very important	1051 (33.7)	795 (37.9)	256 (25.0)	
Rather important	1382 (44.3)	923 (44.0)	459 (44.8)	
Neutral	529 (16.9)	317 (15.1)	212 (20.7)	
Rather unimportant	137 (4.4)	51 (2.4)	86 (8.4)	
Very unimportant	24 (0.8)	12 (0.6)	12 (1.2)	
FVC/day *^a^*				<0.001
≥5 servings *^b^*	256 (7.9)	182 (8.4)	74 (6.9)	
3–4 servings	1210 (37.5)	946 (43.7)	264 (24.8)	
1–2 servings	1709 (52.9)	1016 (46.9)	693 (65.1)	
None	55 (1.7)	21 (1.0)	34 (3.2)	
Cooking: Self-catering/week				<0.001
Daily	611 (18.9)	453 (20.9)	158 (14.9)	
5–6 times	743 (23.0)	526 (24.3)	217 (20.4)	
3–4 times	958 (29.7)	667 (30.8)	291 (27.4)	
1–2 times	744 (23.1)	451 (20.9)	293 (27.6)	
Not at all	170 (5.3)	66 (3.1)	104 (9.8)	
Eating during stressful periods				<0.001
Somewhat/significantly less	1060 (32.8)	700 (32.3)	360 (33.8)	
Unchanged	738 (22.8)	363 (16.8)	375 (35.2)	
Somewhat/significantly more	1435 (44.4)	1104 (50.9)	331 (31.1)	
Sufficient water intake				NS
Yes *^c^*	818 (25.5)	558 (25.9)	260 (24.5)	
No	2396 (74.5)	1596 (74.1)	800 (75.5)	
Both healthy diet recommendations				NS
Yes	115 (3.6)	85 (4.0)	30 (2.8)	
No	3092 (96.4)	2065 (96.0)	1027 (97.2)	

FVC: fruit and vegetable consumption, *^a^* average number of servings; *^b^* international recommendation; *^c^* national recommendation; *p*-values based on chi-squared test.

**Table 4:** Student sleep quality by sex.

Characteristic	Total	Female	Male	*p*-Value
	*N* (%)	*N* (%)	*N* (%)	
Overall sleep quality				NS
Not impaired	1860 (57.7)	1227 (56.8)	633 (59.7)	
Impaired	1363 (42.3)	935 (43.2)	428 (40.3)	
Sleep latency (hours)				0.046
0	892 (27.7)	609 (28.2)	283 (26.6)	
1–2	1414 (43.9)	912 (42.2)	502 (47.3)	
3–4	613 (19.0)	430 (19.9)	183 (17.2)	
5–6	304 (9.4)	210 (9.7)	94 (8.9)	
Sleep duration (hours)				NS
>7	1576 (49.0)	1093 (50.6)	483 (45.7)	
6–7	1439 (44.7)	938 (43.4)	501 (47.4)	
5–6	164 (5.1)	106 (4.9)	58 (5.5)	
<5	40 (1.2)	24 (1.1)	16 (1.5)	
Sleep efficiency (%)				NS
>85	2193 (68.1)	1444 (66.8)	749 (70.8)	
75–84	741 (23.0)	509 (23.6)	232 (21.9)	
65–74	206 (6.4)	151 (7.0)	55 (5.2)	
<65	79 (2.5)	57 (2.6)	22 (2.1)	
Sleep disturbances *^a^*				<0.001
0	343 (10.7)	197 (9.1)	146 (13.8)	
1–6	2569 (79.9)	1725 (79.9)	844 (79.8)	
7–12	297 (9.2)	230 (10.7)	67 (6.3)	
>12	8 (0.2)	7 (0.3)	1 (0.1)	
Daytime dysfunction *^a^*				<0.001
0	312 (9.7)	191 (8.8)	121 (11.4)	
1–2	1615 (50.1)	1047 (48.4)	568 (53.5)	
3–4	1067 (33.1)	754 (34.9)	313 (29.5)	
5–6	230 (7.1)	171 (7.9)	59 (5.6)	

*^a^* number of times per previous week; *p*-value based on chi-squared test.

**Table 5:** Student alcohol, tobacco and substance use by sex.

Characteristic	Total	Female	Male	*p*-Value
	*N* (%)	*N* (%)	*N* (%)	
Alcohol				
Hazardous alcohol consumption				NS
No	2048 (63.3)	1389 (64.0)	569 (61.8)	
Yes	1187 (36.7)	780 (36.0)	407 (38.2)	
My alcohol consumption is harmless				<0.001
Fully applies	1932 (60.6)	1411 (66.2)	521 (49.4)	
Rather applies	679 (21.3)	431 (20.2)	248 (23.5)	
Rather does not apply	330 (10.4)	167 (7.8)	163 (15.5)	
Does not apply at all/cannot judge	247 (7.7)	124 (5.8)	123 (11.7)	
Smoking				<0.001
No	2915 (90.2)	1987 (91.8)	928 (86.9)	
Occasional (not daily)	161 (5.0)	88 (4.1)	73 (6.8)	
Regular (daily)	157 (4.9)	90 (4.2)	67 (6.3)	
Have used substances *^a^*				NS
No	2795 (86.4)	1888 (87.0)	907 (85.0)	
Yes	441 (13.6)	281 (13.0)	160 (15.0)	
Thought about using substances *^a^*				NS
No	2186 (78.3)	1460 (77.5)	726 (80.0)	
Yes	605 (21.7)	423 (22.5)	182 (20.0)	

*^a^* to cope with study demands; *p* values based on chi-squared test.

**Table 6:** Student health behavior and lifestyle characteristics by level of academic study.

Characteristic	BSc	MSc	PhD	*p ^a^*	*p ^b^*	*p ^c^*
	*N* (%)	*N* (%)	*N* (%)			
Physical activity Achievement of recommendations for						
aerobic PA *^d^*	1789 (92.0)	853 (91.8)	372 (87.1)	NS	0.011	0.030
sedentary behavior *^e^*	1092 (56.2)	553 (59.5)	215 (50.4)	NS	NS	0.009
muscle strengthening exercises *^f^*	588 (29.8)	291 (31.3)	169 (39.6)	NS	NS	NS
all types of PA *^g^*	375 (19.3)	187 (20.1)	72 (16.9)	NS	NS	NS
self-rated fitness (very good/good)	422 (21.7)	195 (21.0)	82 (19.2)	NS	NS	NS
Nutrition						
Healthy eating (rather/very important)	1393 (76.0)	724 (81.2)	316 (79.2)	0.008	NS	NS
Recommended FVC *^h^*	149 (7.8)	65 (7.1)	42 (10.1)	NS	NS	NS
Recommended water intake *^i^*	492 (26.0)	225 (24.8)	101 (24.3)	NS	NS	NS
Both healthy diet recommendations *^h, i^*	69 (3.7)	24 (2.6)	22 (5.3)	NS	NS	0.031
Eating during stressful periods (significantly more/less)	485 (25.5)	214 (23.4)	88 (21.1)	NS	NS	NS
Sleep quality						
Impaired sleep quality	854 (45.0)	365 (40.1)	144 (34.7)	0.028	0.001	NS
ATOD						
Risky alcohol consumption	730 (38.3)	320 (35.0)	137 (32.9)	NS	NS	NS
My alcohol consumption is harmless (rather/fully applies)	1549 (82.6)	731 (81.3)	331 (80.1)	NS	NS	NS
Prevalence of non-smokers	1692 (89.0)	835 (91.5)	388 (92.8)	NS	0.016	NS
Have used substances *^j^*	273 (14.3)	127 (13.9)	41 (9.8)	NS	0.032	NS
Thought about using substances *^j^*	385 (23.6)	162 (20.6)	58 (15.3)	NS	0.002	NS

*^a^* BSc vs MSc, *^b^* BSc vs. PhD, *^c^* MSc vs. PhD; *^d^* ≥150 min MPA or ≥75 min VPA or MVPA equivalent; *^e^* ≤8 h/day; *^f^* ≥2 days/week, ≥10 min each; *^g^* aerobic PA guideline and SB recommendation ≤8 h/day and MSE guideline; *^h^* ≥5 servings/day; *^i^* ≥30 mL/kg body mass; *^j^* to cope with study demands; statistical analyses based on binary logistic regression analyses adjusted for sex and with Bonferroni adjustments for multiple group comparisons.

## Data Availability

The data presented in this study are available on reasonable request from the first author (C.M.).
